# Report of a Remarkable Case of Somnolency

**Published:** 1848-06

**Authors:** James Edwards

**Affiliations:** M. D., L.R.C.S. Edin., Forfar


*Report of a remarkable case of Somnolency.* By JAMES EDWARDS, M. D., L.R.C.S. Edin., Forfar.—There lived in the parish of Cortachy, in the county of Forfar, between 1819 and 1834, a certain female, Euphemia Lindsay commonly known through the most of Forfarshire by the appellation of Sleeping Effie. Her peculiarities were as remarkable as those of any one who has appeared in the annals of human history. She was addicted to wandering, and commonly left her home about the time when other people retired to rest, and during the night would frequently wander from twelve to fifteen miles. It was remarked, that when she took these nocturnal journeys, she was sure shortly afterwards to fall into sleeping fits; and it was no unusual thing for her to sleep two or three weeks without awaking. In the winter of 1820 she slept five weeks, and during the spring of 1825 she slept six weeks and three days, which was the longest sleep she had been known to take. This happened during the ministry of the late Rev. John Gourlay, whose son William was a surgeon, and frequently visited her during her somniferous periods. Mr. William Shaw, private teacher, was present at some of Mr. Gourlay's visitations, when he applied singed feathers to her nostrils; and at his request he applied them in such a manner, that the flame touched her nose and face during the time of burning, but she never showed the least symptom of feeling; and to put suspicion beyond doubt, her neighbours frequently put private marks on her bedclothes when or before they retired to rest, and in such a way, that if she had made the smallest stir in the course of the night, the marks must have been removed, but never in the morning, when inspected, had they deviated from where they were placed.

During one of her sleeping fits, it was ascertained that she had taken a drink of water during the night, it being placed close to her bedside; but at no time could it be observed by any of the neighbours who from time to time frequently visited her, that ever she had touched her store of provisions during the time she slept. The last sleeping fit she had came on in the summer season of 1834, and lasted three weeks, and she died of an exhausted constitution three days after she awoke from it, at the age of fifty-six years.

These facts can be authenticated by many respectable people in the parish where she lived, who knew her well, and are still in existence, several of them being relations of her own. During the winter of 1828, I treated her for a neuralgic affection in the region of the heart.—*Lancet.*

